# Tacrolimus inhibits CVB3-targeted regulation of TFEB by PPP3/calcineurin

**DOI:** 10.3389/fcimb.2026.1826524

**Published:** 2026-07-08

**Authors:** Hui Ji, Shanhui Yuan, Wenmin Hu, Yuntao Lu, Jingwen Niu, Xu Hou, Zhenyu Li, Hui Tang

**Affiliations:** 1 Stem Cell Clinical Research Center, Provincial Hospital of Shandong First Medical University, Jinan, China; 2 Department of Pharmacy, Shandong Provincial Hospital Affiliated to Shandong First Medical University, Jinan, China; 3 Clinical Pharmacy Department, Laixi People’s Hospital, Qingdao, China

**Keywords:** autophagy, CVB3, PPP3/calcineurin, tacrolimus, TFEB, virus replication

## Abstract

Recent evidence indicates that Coxsackievirus B3 (CVB3) infection, a common cause of viral myocarditis, triggers the nuclear translocation of transcription factor EB (TFEB) through a mechanism dependent on the serine/threonine phosphatase Protein Phosphatase 3 (PPP3)/calcineurin, independent of its cleavage. Tacrolimus (TAC), a calcineurin inhibitor widely used in immunosuppressive therapy for cardiovascular conditions such as myocarditis and post-transplant vasculopathy, may modulate TFEB activity in this context. This study investigated the effect of TAC on TFEB regulation during CVB3 infection in HeLa cells. Our results demonstrate that TAC significantly suppresses both the nuclear accumulation and transcriptional activity of TFEB. Conversely, knockdown of Protein Phosphatase 3 Catalytic Subunit (PPP3C) enhances TFEB protein expression and its nuclear localization, indicating that TAC calcineurin-dependent mechanism beyond simple enzymatic inhibition. Moreover, TAC similarly inhibits the nuclear expression and transcriptional activity of both Δ60-TFEB (a cleavage fragment lacking the first 60 amino acids) and TFEBQS60LP (cleavage-resistant mutant). Knockdown of PPP3C leads to increased nuclear distribution of these TFEB variants, confirming that TAC targets PPP3/calcineurin to regulate TFEB and its modified forms. These findings suggest that TAC interferes with the CVB3-induced activation of TFEB, thereby influencing cellular autophagy and viral replication. Importantly, TAC treatment attenuates CVB3-induced autophagic response and reduces viral protein expression and RNA levels in infected cells. Collectively, this study reveals a novel role for TAC in modulating TFEB subcellular localization and activity in the context of CVB3 infection, with potential implications for the pharmacological management of viral myocarditis and associated cardiovascular pathologies.

## Introduction

Autophagy is a cellular degradation pathway that degrades cytoplasmic components such as damaged proteins and organelles in the cell, thus playing a key role in cellular homeostasis (Alexandre and Jörn, 2022). TFEB is a key factor in the regulation of autophagy and lysosomal function and is involved in a variety of cellular regulations, regulating autophagy by promoting the formation of autophagosomes and the fusion of autophagosomes and lysosomes (Carmine et al., 2013). Recently, a new pathway has been discovered to mediate TFEB, and dephosphorylation of TFEB is mediated by the phosphatase PPP3/calcineurin (L, 2021) Under certain physiological or pathological conditions, Ca^2+^ is released into the cytoplasm via the calcium channel protein MCOLN1 (mucolipin1). In this case, Ca^2+^ rises in the cytoplasm and PPP3/calcineurin is activated to induce dephosphorylation of TFEB nuclear translocation and transcription of downstream target genes, which correspondingly increases lysosome fusion with the plasma membrane, enhances cytoplasmic solidification of lysosomes, and improves cell clearance (Kihyoun and MyungShik, 2022; Zhiyuan et al., 2023).

TAC is a widely used clinical inhibitor of PPP3/calcineurin, and its immunological mechanism is mainly through binding to FKBP12 in the cytoplasm to form a complex, which inhibits the dephosphorylating activity of PPP3/calcineurin and exerts an immunosuppressive effect (Gabriela et al., 2017). Additionally, it is reported that TAC promoted TFEB translocation and autophagy levels (Dongyoung et al., 2017; qing et al., 2020). While others have reported that TAC induces down-regulation of TFEB transcript levels and reduces the expression of lysosome-associated membrane proteins (Zhensheng et al., 2023; Gao et al., 2025; Ma et al., 2025). From these, it can be seen that the mechanism of TAC-mediated regulation of autophagy by TFEB has not yet been fully elucidated.

Coxsackievirus B3 (CVB3) belongs to the genus Enterovirus in the family Picornavridae (Uk et al., 2022). Our previous study showed that TFEB was cleaved by CVB3 viral protease 3C, and that TFEB was cleaved by 3C at a position behind N-terminal glutamine 60 (Yasir et al., 2021). Studies performed on cleaved TFEB (Δ60-TFEB) revealed that it retained the ability to homo- and heterodimerise members of the basic helix-loop-helix-leucine-zip (bHLH-Zip) family of MITF/TFE transcription factors and the ability to bind CLEAR (Coordinated Lysosomal Enhancement And Regulation) elements. For the TFEBQS60LP, despite resistance to viral protease 3C cleavage, TFEBQS60LP was significantly translocated into the nucleus after infection, and under si-PPP3C, PPP3C protein expression was reduced, CVB3 induced a decrease in the intranuclear content of TFEBQS60LP. In this study we focused on 1:the mechanism of TAC in targeting and regulating TFEB, Δ60-TFEB and TFEBQS60LP; and 2:the role of TAC in targeting and regulating TFEB by CVB3 and the related mechanism.

## Materials and methods

### Cell culture and cell transfection

HeLa cells were cultured with DMEM medium containing 10% FBS (Gibco,10270-106) in a 5% CO_2_ incubator (37 °C), and passaged for inoculation when the cell confluence reached 80% to 90%. In our previously published study, cell viability was assessed by CCK-8 assay after treatment with TAC at concentrations of 5, 10, 20, 40, 80, and 120 μM for 24 h. Compared with the blank control group, the OD450 values showed no significant changes following treatment with different concentrations of tacrolimus, indicating that tacrolimus exhibited no cytotoxicity within the concentration range tested in this experiment (Hu et al., 2025)., and the results showed that the different concentrations of TAC significantly reduced TFEB expression (**Supplementary Figures 1A–C**). We finally selected 20μM and 40μM for subsequent experiments. It should be noted that the TAC concentrations used in this *in vitro* setting exceed therapeutic plasma levels in patients (typically in the low nanomolar range). Such higher concentrations are commonly required in cell−culture studies to drive the formation of the FKBP12–TAC–calcineurin ternary complex and to compensate for the absence of pharmacokinetic distribution factors. and comparable concentrations have been used in published investigations of calcineurin−dependent autophagy regulation (Medina et al., 2015; Gao et al., 2025).

Homozygous HeLa cells in logarithmic growth phase were taken and inoculated into 12-well plates. After culturing for 2–4 days, cells at 70-90% confluence were used for transfection. Lipofectamine 3000 (Invitrogen,L300008) was used to transfect TFEB-GFP, TFEBQS60LP-GFP, Δ60-TFEB-GFP cells to construct the cell line (refer to the instruction manual of the lipofectamine 3000 reagent). Subsequent experiments were performed after 24h of intervention.

### Western blot

Proteins were extracted from whole-cell using pre-cooled RIPA lysate and 1% protein phosphatase inhibitor. After protein concentration was determined by BCA method, 30μg of protein samples were separated by electrophoresis through 10% SDS-PAGE. The proteins were transferred to a PVDF membrane (Millipore, IPVH00010) and blocked with 5% no-fat milk (Solarbio, D8340). Primary antibodies were then used: TFEB (Cell Signaling Technology, D2O7D), GFP (Sigma Aldrich, SAB5300167), PPP3C/calcineurin (Santa Cruz Biotechnology, sc-17808), LC3B (NOVUS, NB100-2220SS), ACTB (Abclonal, AC026); on day 2, the membranes were washed three times with TBST (10 min each time) and then incubated with horseradish peroxidase (HRP)-coupled secondary antibody IgG (H+L) (Abclonal, AS014) for 1 h at room temperature. Finally, the membranes were incubated with chemiluminescence (ECL) reagent (Biosharp, BL520A) to visualize and quantify the antibody complexes.

### Quantitative real-time PCR

Total RNA was extracted using the RNeasy Mini kit (Qiagen, 74104). The amount of RNA was assessed spectrophotometrically using the NanoDrop-1000. RNA was reverse transcribed into cDNA according to the instructions of the AG kit (Accurate Biology, AG11728). cDNA was diluted at 1:1000 for RT-qPCR amplification after reverse transcription. The conditions were 95 °C for 30 s, followed by denaturation at 95 °C for 5 s, 55 °C for 30 s, and 72 °C for 30 s for a total of 40 cycles (Accurate Biology, AG11739). GAPDH was used for standardization of the assay. The experiment was performed in triplicate. The relative expression was calculated using the 2^-ΔΔCt^ method. Primer sequences are detailed in **Table 1**.

**Table 1:** Primer sequences used for quantitative PCR.

Gene	Forward primer	Reverse primer
ATP6V1H-Human	CCCTGAAGAGAAGCAAGAGATG	TGCAGCATATCATCCACCATAG
MCOLN1-Human	GGAAAGCAGCTCCAGTTACA	GATGAGGCTCTGGAGGTTAATG
CTSB-Human	GGACAAGCACTACGGATACAA	GTAGAGCAGGAAGTCCGAATAC
M6PR-Human	CTCAGTGTGGGTTCCATCTTAC	GGGAAACTGCTCCATTCCTT
RAB7A-Human	CCTGGAGTCTTGGCCATAAAG	GAGAAGGTCCAAGTTCTGGTTC
PPP3CA-Human	GCTGCCCTGATGAACCAACA	GCAGGTGGTTCTTTGAATCGG

### Immunofluorescence and confocal microscopy

HeLa cells were inoculated into 12-well plates with coverslips (2.5×10^4^ cells/well), and after TAC intervention for 24 h, the medium was aspirated and washed three times with PBS. 4% paraformaldehyde fixed the cells for 20 min, and washed three times with PBST. 0.1% Triton X-100 (thermo, HFH10) treated the cells for 20 min at room temperature, and washed three times with PBS. 5% BSA (Solarbio, H1130) was blocked at room temperature for 1 h. Then primary antibodies TFEB (Santa Cruz Biotechnology, sc-166736), anti-GFP (Santa Cruz Biotechnology, sc-9996), LC3B (Santa Cruz Biotechnology,sc-376404), LAMP1 (Santa Cruz Biotechnology, sc20011), and incubated overnight at 4 °C. After aspirated the primary antibody, a fluorescent secondary antibody (Abcam, ab150115) was added, and incubated at room temperature away from light for 1 h. Finally, washed six times with PBST (5 min each time). The slices were sealed with DAPI sealer (sigma, F6057), and images were captured using a laser confocal ortho-microscope (Leica) with a 100x objective lens.

Nuclear localization of TFEB was determined by co-staining with DAPI. The nuclear fluorescence intensity of TFEB (or GFP-tagged variants) was quantified using ImageJ by outlining DAPI-positive regions as the nuclear area and measuring mean fluorescence intensity in at least 50 cells per condition from three independent experiments. Due to the use of whole-cell lysates, Western blot analysis reflects total cellular TFEB levels; subcellular distribution was determined by confocal immunofluorescence.

### 4× CLEAR luciferase assay

4× CLEAR luciferase reporter (Addgene, 66800) and Renilla control luciferase plasmid (Addgene, 87121) were cotransfected with Lipofectamine 3000 (Invitrogen, L300008). 20μL of Luciferase Assay reagent (Promega, E2920) was added 48h later and shaken to mix. Immediately after mixing, the firefly luciferase fluorescence values were measured used a luciferase marker (Berthold), followed by the addition of 20μL of Stop&Glo^®^ reagent, shook and mixed, and then left to stand for 3 min before detected the sea kidney luciferase fluorescence values using an enzyme marker. Finally calculated the ratio of Firefly luciferase activity to Renilla luciferase activity. The experiment was carried out three times and the average value was taken.

### Plasmids and small interfering RNA

pEGFP-N1-TFEB (Addgene,38119), Δ60-TFEB and TFEBQS60LP plasmid were generated in the Key Gen Bio TECH. 60-TFEB was synthesized by deleting the first 60 amino acids (retaining the start codon) using the synthetic overexpression plasmid pEGFP-N1-TFEB as a template. TFEBQS60LP used the synthesized overexpression plasmid pEGFP-N1-TFEB as a template to mutate the Q.S amino acid at position 60.61, to the L.P amino acid. The sense and antisense sequences of the siRNAs used in this study are listed in **Table 2**.

**Table 2:** Sense and antisense siRNA sequences.

Gene	Sequence (5’to 3’)
PPP3C siRNA sense strand	UCACAGAGAUGCUGGUAAATT
PPP3C siRNA antisense strand	U/UUACCAGCAUCUCUGUGATT
Control siRNA sense strand	GUAUGACAACAGCCUCAAGTT
Control siRNA antisense strand	CUUGAGGCUGUUGUCAUACTT
TFEB siRNA sense strand	GCAUCAAGGAGUUGGGAAUTT
TFEB siRNA antisense strand	AUUCCCAACUCCUUGAUGCTT
Control siRNA sense strand	GUAUGACAACAGCCUCAAGTT
Control siRNA antisense strand	CUUGAGGCUGUUGUCAUACTT

### CVB3 virus titration

HeLa cells in the logarithmic growth phase were seeded into a 96-well plate the day before the assay to ensure even distribution. Ten sterile microcentrifuge tubes were prepared, each containing 90 μL of CVB3 maintenance medium (DMEM supplemented with 2% FBS). A 10 μL aliquot of the CVB3 virus stock was added to the first tube, mixed thoroughly, and then 10 μL from this dilution was transferred to the second tube. Serial ten-fold dilutions were performed in this manner, yielding a total of 10 different virus dilutions. The culture medium was removed from the 96-well plate, and 90 μL of each virus dilution was added to the wells, with 8 replicate wells per dilution. The plate was incubated at 37 °C in a 5% CO_2_ incubator overnight. The cells were observed daily under a light microscope for the presence of cytopathic effect (CPE) until no further changes were observed in the wells. The number of wells exhibiting CPE at each dilution was recorded, and the 50% tissue culture infectious dose (TCID_50_) was calculated according to the Reed–Muench method. **The CVB3 stock titer was determined to be 10^5^ TCID_50_/mL.**

### CVB3 infection

HeLa cells in the logarithmic growth phase were rinsed three times with PBS when the cell fusion rate reached 80%–90%. The CVB3 stock (Nancy strain, donated by Shandong Academy of Medical Sciences) had a titer of 10^5^ TCID_50_/mL. For infection, the virus inoculum was diluted in DMEM containing 2% fetal bovine serum (CVB3 maintenance solution) to achieve a multiplicity of infection (MOI) of approximately 1, based on the cell number at the time of inoculation. The infected cultures were incubated at 37 °C in 5% CO_2_. CPE was observed daily under a light microscope. For TAC treatment combined with CVB3 infection, cells were pretreated with 20 or 40 μM TAC in complete medium for 24 h prior to viral inoculation. After removal of the TAC-containing medium, cells were infected with CVB3 at the indicated MOI in the presence of the same concentration of TAC and maintained for the duration of infection (5 h).

### Statistical analyses

The combined data obtained from the above experiments were statistically analyzed using GraphPad Prism (GraphPad Software, USA), and three or more independent technical replicates were performed for each experiment. Comparisons between groups were made using one-way ANOVA (one-way ANOVA), with a statistically significant difference of P<0.05.

## Results

**Figure 1:**
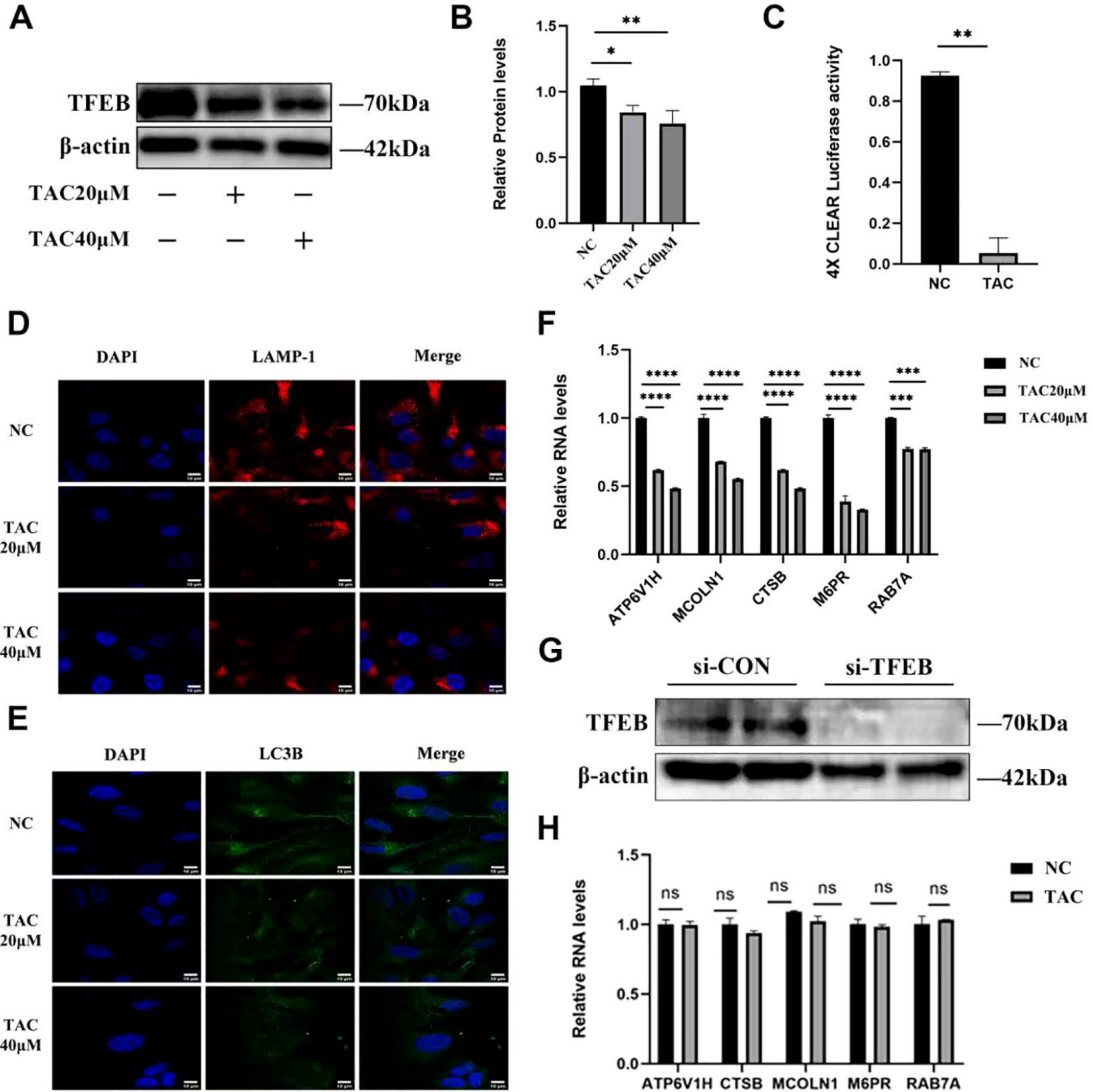
TAC inhibits TFEB expression, transcriptional activity, and autophagy-lysosomal markers. TAC inhibits. Western blot detection of TFEB. **(A, B)** 4× CLEAR Luciferase reporter gene assay for TFEB transcriptional activity. **(C)** LC3B and LAMP-1 staining results. Scale bar=10μm. **(D, E)** RT-qPCR detection of TFEB downstream target gene transcript levels. **(F)** TFEB gene was knocked down and the transcript levels of TFEB downstream target genes were detected. n = 3-4 per group. **(G, H)** Data are shown as the mean±SEM. *P<0.05, **P<0.01, ***P<0.001, ****P<0.0001. ns, not significant.

After TAC treatment, the expression of TFEB was reduced (**Figures 1A, B**) and the 4×CLEAR Luciferase activity was significantly reduced in TAC-treated cells (**Figure 1C**). Immunofluorescence results showed that autophagy marker LC3B puncta/signal were reduced by TAC and the expression of lysosome-associated membrane protein LAMP-1 was significantly lower after TAC treatment (**Figures 1D, E**). In addition, the downstream target genes of TFEB, including M6PR (mannose 6-phosphate receptor) (Yasir et al., 2021) CTSB (cathepsin B) (Yasir et al., 2021; Dan et al., 2023),ATP6V1H (ATPase H+ transporter V1 subunit H) (L et al., 2017; Araujo et al., 2018), and MCOLN1 (mucolipin 1) (Dan et al., 2023) were down-regulated by TAC (**Figure 1F**).

Meanwhile, we employed siRNA technology, knocked down TFEB protein, and used RT-qPCR to detect the effect of TFEB gene silencing on autophagy-related gene expression. The results showed that there was no obvious trend of change in downstream gene expression after TFEB knockeddown.(**Figures 1G, H**).

**Figure 2:**
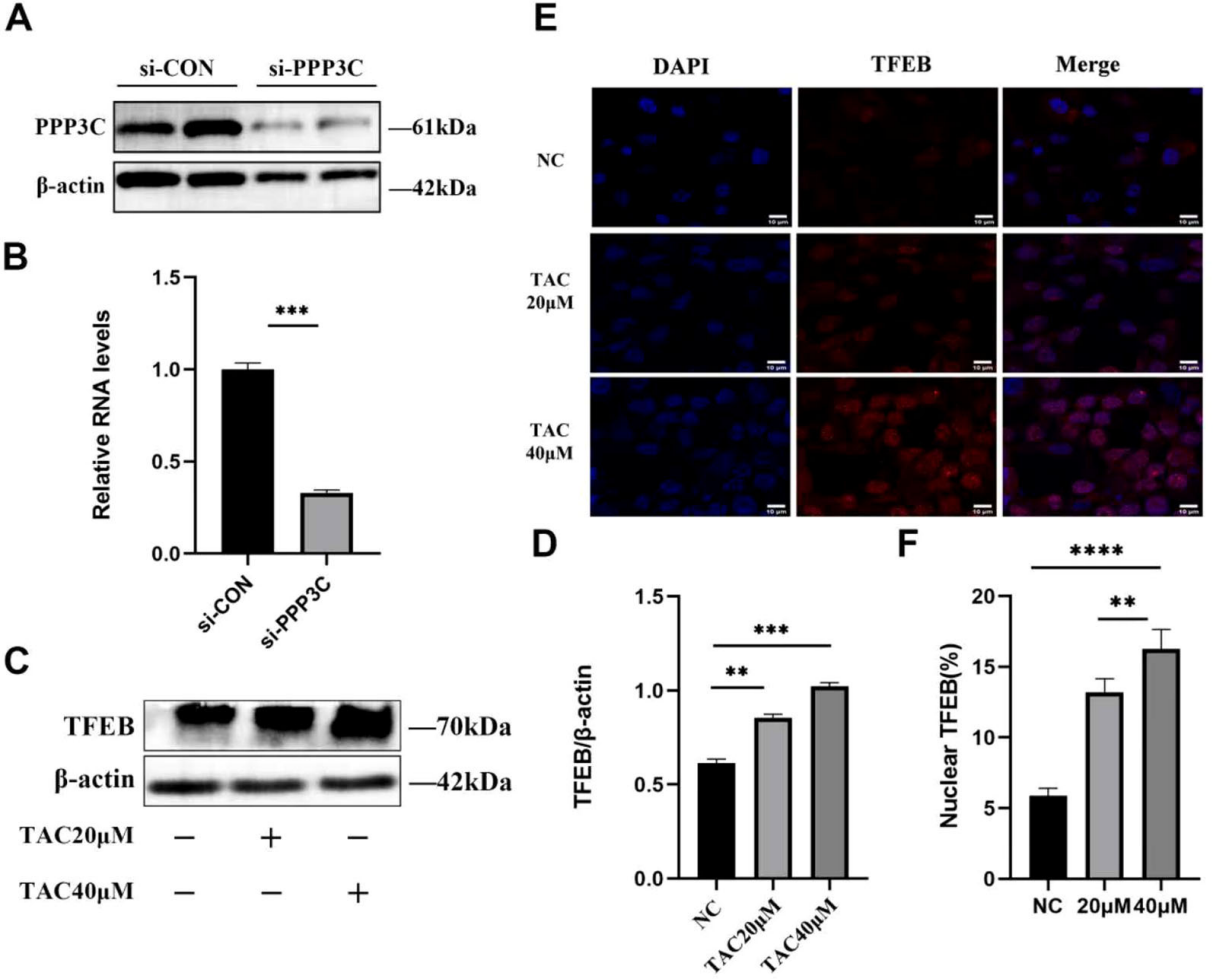
TAC inhibits TFEB by targeting PPP3/calcineurin. **(A, B)** Western blot and RT-qPCR validated PPP3C Silence levels. **(C, D)** PPP3C knocked down, western blot was performed to detect the expression levels of TFEB. **(E, F)** PPP3C knocked down, then different concentrations of TFEB in the nuclear. Scale bar=10μm. n = 3-4 per group. Data are shown as the mean±SEM. **P<0.01, ***P<0.001, ****P<0.0001.

In order to further determine whether PPP3C is a target protein of TAC-regulated TFEB, we used siRNA technology to knock down PPP3C (**Figures 2A, B**). The results showed that TFEB protein expression was elevated, and in addition autophagy marker LC3II/LC3I ratio was also increased under TAC-induced (**Figures 2C, D**). Similarly, immunofluorescence showed that after knocked down PPP3C protein, the nuclear localization of TFEB in the TAC group was more concentrated than that in the control group and the intranuclear expression was also significantly increased (**Figures 2E, F**).

**Figure 3:**
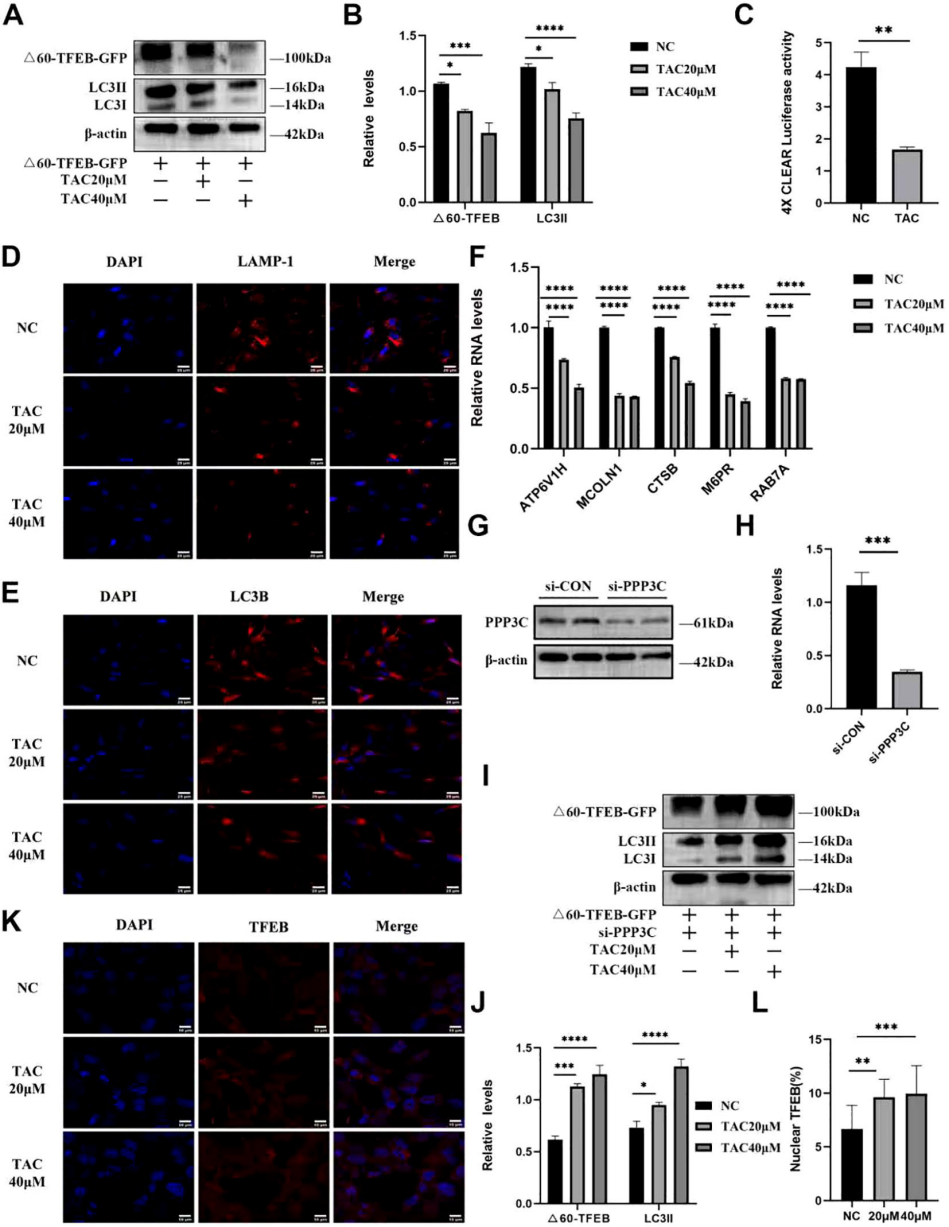
TAC inhibits Δ60-TFEB and TFEBQS60LP by targeting PPP3/calcineurin. TAC inhibits Δ60-TFEB and TFEBQS60LP by targeting PPP3/calcineurin. **(A, B)** Western blot was performed to detect the expression level of Δ60-TFEB and LC3II. **(C)** 4× CLEAR Luciferase reporter gene assay for Δ60-TFEB transcriptional activity. **(D, E)** Immunofluorescence detection of LC3B and LAMP-1 staining. Scale bar=25μm. **(F)** The level of transcription of TFEB downstream target genes by RT-qPCR. **(G, H)** Western blot and RT-qPCR validated PPP3C Silence levels. **(I, J)** PPP3C knocked down, western blot was performed to detect the expression levels of ∆60-TFEB and LC3II. **(K, L)** PPP3C knocked down, then different concentrations of Δ60-TFEB in the nuclear. Scale bar=10µm. n = 3-4 per group. Data are shown as the mean ± SEM. * P<0.05, ** P<0.01, *** P<0.001, **** P<0.0001.

In order to determine whether TAC could regulate Δ60-TFEB and TFEBQS60LP, we constructed Δ60-TFEB-GFP and TFEBQS60LP-GFP transfected cell lines. Western blotting results showed that TAC treatment decreased the expression of Δ60-TFEB (**Figures 3A, B**) and TFEBQS60LP(**Figures 3A, B**). Meanwhile, the protein expression of LC3-II also showed a decreasing trend (**Figures 3A, B**). Additionally, the 4×CLEAR luciferase assay showed that TAC inhibited the transcriptional activity of Δ60-TFEB (**Figure 3C**) and TFEBQS60LP (**Figure 3C**), which was consistent with the similar result of the expression of autophagy-related genes in the downstream of Δ60-TFEB and TFEBQS60LP (**Figure 3F**).

Moreover, TAC treatment appeared to reduce autophagosomes as LC3B punctae and LAMP-1 were reduced (**Figures 3D, E**). After knocked down PPP3C (**Figures 3G, H**), Δ60-TFEB and LC3II were increased in the TAC-treated group compared with the control group (**Figures 3I, J**). Immunofluorescence results showed that knocked down PPP3C allowed TFEB to aggregate toward the nucleus and enhanced intranuclear expression (**Figures 3K, L**). Similarly, TFEBQS60LP showed the same result (**Figures 3E–H**).

**Figure 4:**
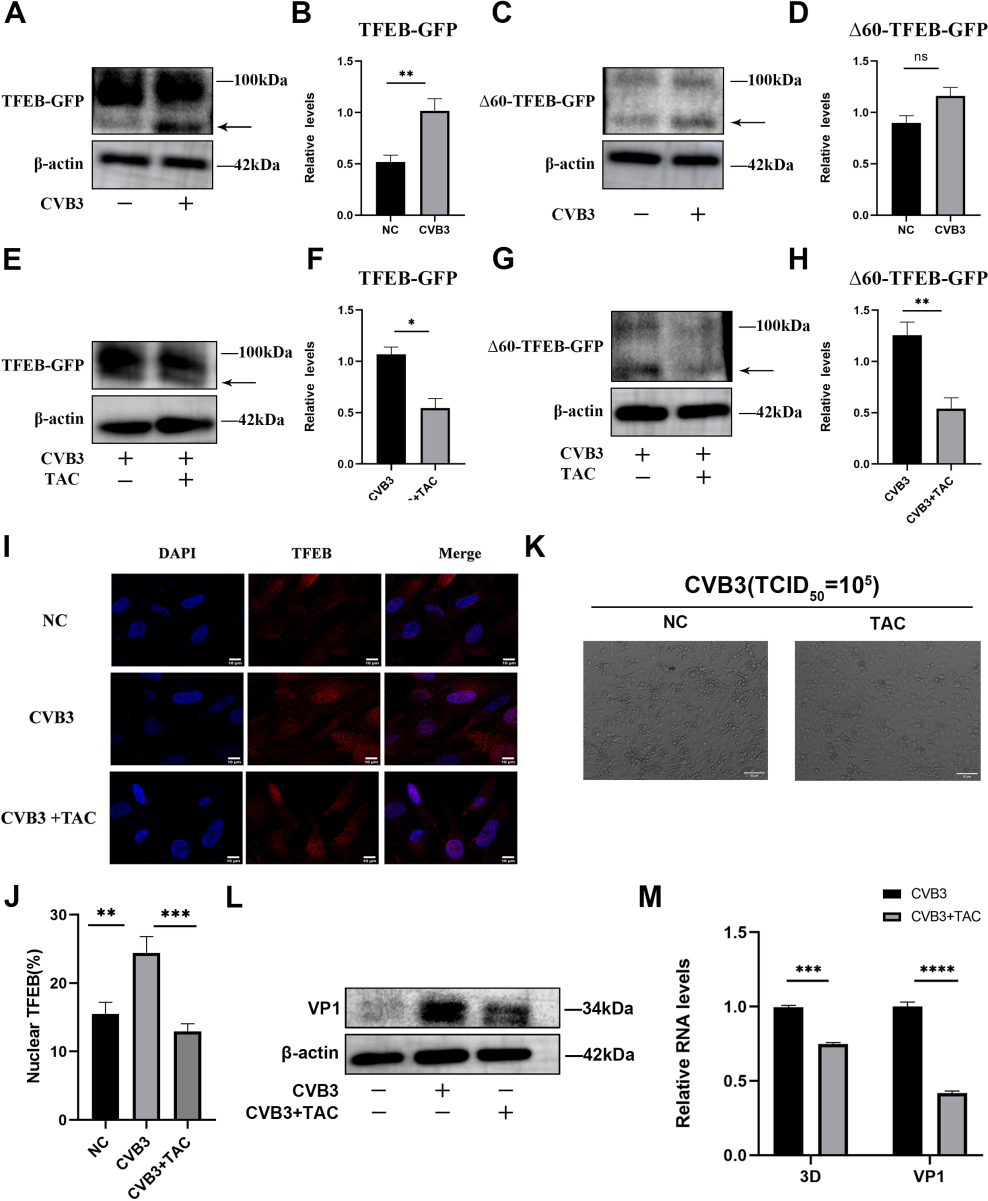
TAC plays a potential role in CVB3-targeted regulation of the TFEB process. **(A–D)** Western blot was performed to detect the expression level of TFEB and ∆60-TFEB with CVB3 infection 5h. **(E–H)** Western blot was performed to detect the expression level of TFEB and ∆60-TFEB with CVB3 and TAC infection. **(I, J)** Immunofluorescence detection of TFEB and ∆60-TFEB staining with CVB3 and TAC infection. **(K)** CPE was observed under microscope at regular intervals. Scale bar=50μm. **(L)** Western blot was performed to detect the expression level of VP1. **(M)** RT-qPCR was performed to detect the transcription of genes of CVB3. n = 3-4 per group. Data are shown as the mean±SEM. *P<0.05, **P<0.01, ***P<0.001, ****P<0.0001. ns, not significant.

To confirm the role of TAC in CVB3-targeted regulation of the TFEB process, CVB3 infection for 5h and detection of TFEB protein expression. The expression of WT-TFEB and 60-TFEB were increased and the emergence of TFEB reactive fragment (**Figures 4A–D**). The same result was obtained with CVB3 infection of TFEB^QS60LP^ (**Figures 4A, B, E**). Moreover, TFEB nuclear expression is centralized after CVB3 infection, but transcript levels were decreased (**Figure 4I, L**). However, TAC reduced CVB3 virus replication (**Figures 4K–M**) and the expression of TFEB (**Figures 4E–H**). Similarly, the same result was obtained with TAC infection of TFEB^QS60LP^ (**Figures 4C–E**).

## Discussion

Autophagy is the process by which cells break down highly conserved proteins or organelles in response to external factors. During this process, cytoplasmic components, such as damaged proteins and organelles, are encapsulated in double-membrane autophagic vesicles that are ultimately transported to the lysosome for degradation (Medina et al., 2015). Amino acids and other small molecules resulting from the degradation process can be reutilized or used to generate energy, which in turn sustains the cell’s basic life activities (W and Liron, 2019). Autophagy plays an important role in the developmental processes of the organism, in human diseases, and in cellular responses to nutrient deficiencies.

TFEB is the most important transcription factor regulating autophagy, regulating the expression of lysosomal and autophagy-related genes, participating in a variety of signal pathways and is controlled by a number of upstream factors, including phosphorylation, ubiquitination and acetylation (Gennaro and Andrea, 2016; Jayanta et al., 2023). TFEB shuttles between the nuclear and lysosomes to regulate cellular metabolism, is generally present in the cytoplasm and enters into the nucleus under stress or starvation to bind to autophagy-related gene promoters of autophagy-related genes (Jianbin et al., 2018). It is currently believed that the main mechanism regulating the activity of TFEB is its phosphorylation (Carmine et al., 2012; Roczniak-Ferguson et al., 2012) which can be phosphorylated by several heavy protein kinases such as mTORC1, MAPK1/ERK2, and protein kinases (Chen et al., 2022; Shengyuan et al., 2022; Thi et al., 2023), Dephosphorylated TFEB directly binds to its promoter, activating the expression of autophagy-phase-associated genes and to promoting lysosomal biosynthesis (Jin et al., 2021; Katarzyna, 2023).

TFEB acts as a switch in the autophagy-lysosome pathway that regulates the expression of autophagy-related genes and accelerates cellular metabolism (Kihyoun et al., 2022).LC3B is the most commonly used autophagy marker (qing et al., 2020). Our results showed that TAC inhibited the transcriptional activity of TFEB. In addition, the down-regulation of TFEB expression level was accompanied by a decrease in the expression of LC3B and LAMP-1. It suggested that TAC impairs lysosomal function by mediating TFEB. TAC-induced down-regulation of TFEB expression was accompanied by decreased transcription levels of downstream target genes. After si-TFEB, TAC-induced autophagy-related genes did not change, which illustrated that the downstream genes of TFEB could be regulated by TFEB itself rather than induced by the external factor TAC. It is reasonable to assume that TFEB is not only a target of autophagy regulation, but also an important switch mediating the autophagy-lysosome pathway.

Medina et al (L, 2021)found that under stress, the calcium channel protein MCOLN1 releases Ca^2+^ into the cytoplasm to activate PPP3/calcineurin, which binds and dephosphorylates TFEB, which enters the nuclear to activate downstream genes, promote transcription and enhance lysosomal catabolism. PPP3/calcineurin has been reported to dephosphorylate TFEB at critical residues and to enhance nuclear translocation and activation of TFEB during starvation or stress conditions (Mateus et al., 2023). In this study, we revealed the mechanism of action of TAC in targeting TFEB. Our results showed that TAC induced a decrease in endogenous and exogenous TFEB expression, a decrease in TFEB nuclear translocation, and a decrease in transcriptional activity. Interestingly, after si-PPP3C, the TAC-induced nuclear localization of TFEB showed a centralized trend in both endogenous and exogenous TFEB (**Figures 2G–J**). TAC-induced exogenous TFEB expression was also elevated after si-PPP3C in exogenous TFEB-GFP transfected cell lines. In this study, we revealed the mechanism of action of TAC in targeting TFEB. Our results showed that TAC induced a decrease in endogenous and exogenous TFEB expression, a decrease in TFEB nuclear translocation, and a decrease in transcriptional activity. Interestingly, after si-PPP3C, the TAC-induced nuclear localization of TFEB showed a centralized trend in both endogenous and exogenous TFEB (**Figures 2G–J**). TAC-induced exogenous TFEB expression was also elevated after si-PPP3C in exogenous TFEB-GFP transfected cell lines. This seemingly paradoxical result—that PPP3C knockdown restores TFEB in the presence of TAC—led us to re−examine the nature of TAC’s action. Rather than simply loss of phosphatase activity, our data indicate that TAC binding converts PPP3C into a dominant−negative scaffold that actively suppresses TFEB. Removal of this gain−of−function scaffold by siRNA relieves the suppression, allowing TFEB to re−accumulate in the nucleus. This interpretation is entirely consistent with the known conformational change induced by FKBP12−TAC−calcineurin complex formation (Mevizou et al., 2024; Samuelsson et al., 2025; Zhou et al., 2025), and with the physical interaction between calcineurin and TFEB that underlies its normal dephosphorylation function. It explains not only the nuclear translocation data but also the observed decrease in total TFEB protein (reflecting cytoplasmic retention and degradation). Thus, the counterintuitive result paradoxically provides strong evidence that TAC regulates TFEB by targeting PPP3C, albeit through a mechanism beyond simple enzymatic inhibition. We therefore assume that PPP3C could be a switch for TAC to regulate TFEB, and TAC inhibited TFEB expression and disrupted the nuclear translocation of TFEB by targeting PPP3/calcineurin, primarily by converting it into a suppressive scaffold rather than solely blocking its dephosphorylation activity.

In our previous study, CVB3’s protease 3C cleaved TFEB and Δ60-TFEB was functionally defective but retained the ability to bind to CLEAR elements and to homo- and heterodimerise members of the bHLH-Zip family of MITF/TFE transcription factors. In this study, we demonstrated that despite the defective function of Δ60-TFEB, TAC could inhibit the expression and transcriptional activity of Δ60-TFEB. After si-PPP3C, the expression and nuclear localization of Δ60-TFEB protein were increased in TAC-treated cells. All data showed that despite defective Δ60-TFEB function, Δ60-TFEB could regulate lysosomal function and autophagy, and TAC could regulate Δ60-TFEB expression and nuclear localization by targeting PPP3/calcineurin. In the mutant TFEB^QS60LP^cell line, TFEB^QS60LP^ could still regulate the expression of lysosomal and autophagy-related genes, and regulate the biosynthesis of autophagy and lysosomal. TAC also inhibited the transcriptional activity of TFEB^QS60LP^ and nuclear localization by targeting PPP3/calcineurin.

In this study, we demonstrated that TFEB expression in the nucleus increased with CVB3 infection, but downstream gene expression decreased, which is consistent with our previous study. Moreover, TAC treatment was associated with reduced CVB3 VP1 protein levels and diminished viral RNA transcript abundance, as evidenced by both western blot and RT-qPCR analyses (**Figures 4L, M**). While these results are suggestive of reduced viral propagation, they do not constitute direct quantitative measures of infectious viral particles. TAC did not, however, impede the phenomenon of CVB3 cleavage. Also, we found that CVB3 infection increased Δ60-TFEB expression but not significantly, presumably, Δ60-TFEB was affected by CVB3 cleavage, and thus the increase in Δ60-TFEB intranuclear expression induced by CVB3 was not obvious. TAC disrupted this pattern, and under CVB3 infection, TAC still reduced the nuclear expression of Δ60-TFEB, showing a decrease after TAC treatment compared to CVB3 induction.

Similarly, TAC still played an inhibitory role in CVB3-targeted regulation of TFEB^QS60LP^. Our previous study showed that PPP3C protein expression was reduced under si-PPP3C, and CVB3 induced a decrease in the intranuclear content of TFEBQS60LP. Combined with the above results, it is reasonable to assume that TAC could also play a role in the CVB3-targeted induction of TFEB by targeting PPP3/calcineurin.

**Figure 5:**
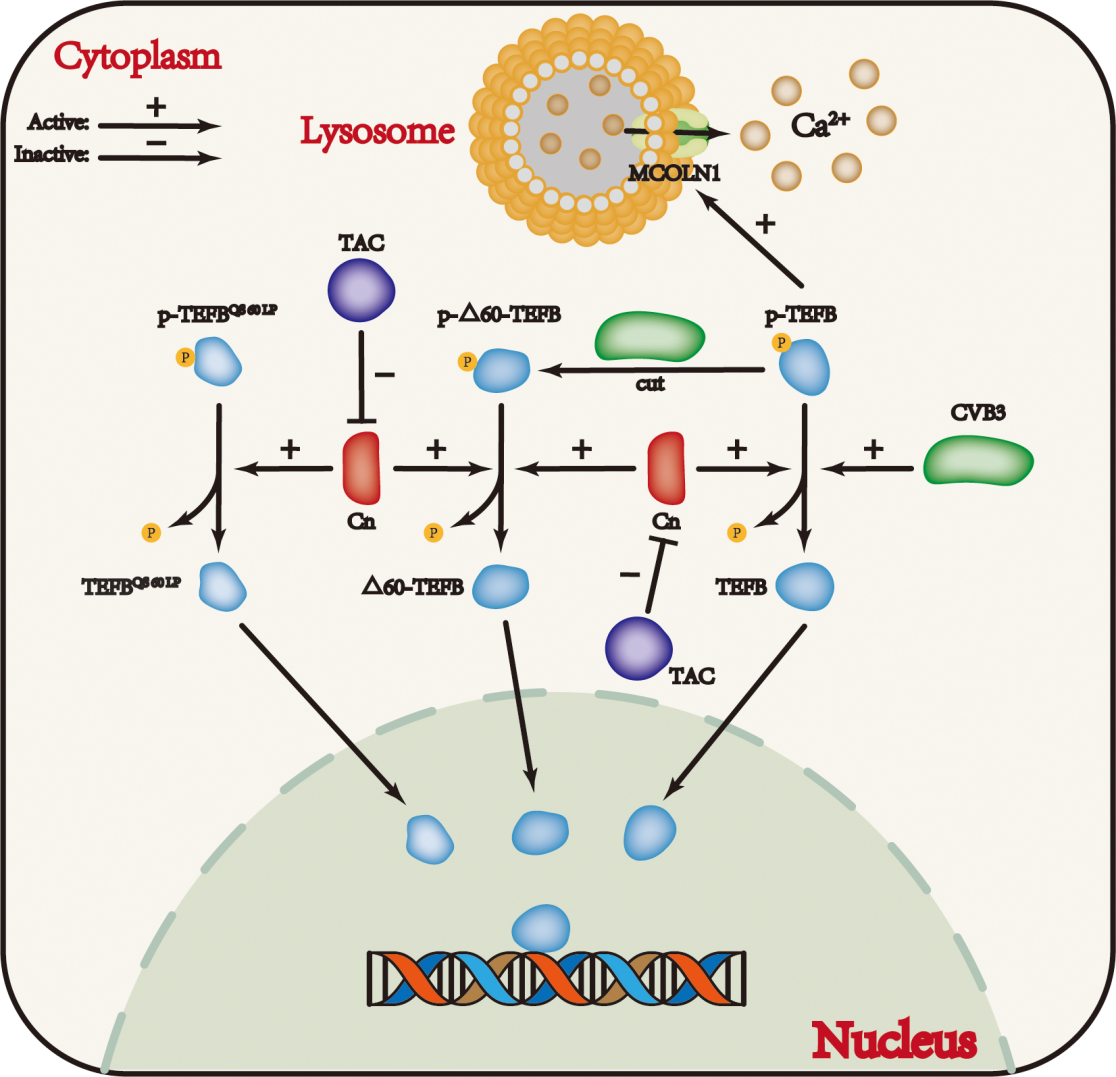
Working model illustrating the mechanism by which TAC inhibits CVB3-mediated regulation of TFEB.

Together, these findings support a model in which TAC targets PPP3/calcineurin to suppress CVB3−induced TFEB nuclear translocation and transcriptional activity (**Figure 5**). It is noteworthy that additional signaling inputs may converge on TFEB regulation in the context of CVB3 infection. Recently, PKR−like endoplasmic reticulum kinase (PERK), a core effector of the unfolded protein response (UPR), has been implicated in the regulation of TFEB nuclear translocation and transcriptional activity (Cai et al., 2025; Singh et al., 2025).Activation of PERK has been shown to promote TFEB dephosphorylation and lysosomal biogenesis under ER stress conditions, providing a mechanistic link between ER homeostasis and autophagy. Moreover, tripartite motif−containing protein 29 (TRIM29) was recently demonstrated to modulate PERK−mediated ER stress immune responses in CVB3−induced viral myocarditis (Junying et al., 2024). In that study, TRIM29 deficiency exacerbated CVB3−induced cardiac inflammation and ER stress, suggesting that TRIM29 plays a protective role by regulating the PERK signaling axis.

In the present study, we observed that TAC inhibits CVB3−induced TFEB nuclear translocation and transcriptional activity through a PPP3/calcineurin−dependent mechanism. However, whether TAC may additionally intersect with the TRIM29−PERK−TFEB pathway remains an open question. Given that TAC is a known calcineurin inhibitor with pleiotropic effects on UPR signaling, it is conceivable that TAC could modulate PERK activity either directly or indirectly, thereby contributing to the inhibition of TFEB. Alternatively, TAC−mediated suppression of PPP3/calcineurin may represent the predominant mechanism in our HeLa cell model, and the contribution of TRIM29−PERK signaling to TFEB regulation in this setting awaits dedicated investigation. Future studies employing PERK−specific inhibitors or activators, combined with TRIM29 overexpression or silencing in cardiomyocyte models, would be valuable to dissect the potential crosstalk between TAC−PPP3/calcineurin and TRIM29−PERK pathways in the regulation of TFEB during CVB3 infection.

Several limitations of this study should be acknowledged. First, the majority of our experiments were conducted in HeLa cells, an epithelial cell line derived from cervical carcinoma. While HeLa cells offer excellent transfection efficiency and have been extensively used as a model system for studying CVB3-host interactions and autophagy regulation (Prevention SKLoID, Control CICfD, Treatment of Infectious Diseases NIfVDC, Prevention CCfDC, Prevention B, China, 2018; Mi et al., 2021; Yasir et al., 2021), they are not of cardiac origin. The relevance of our findings to viral myocarditis remains to be validated in more physiologically relevant models, including primary cardiomyocytes, cardiac-derived cell lines (e.g., H9c2 or HL-1), and ultimately *in vivo* models of CVB3-induced myocarditis. However, it should be noted that the core signaling axis investigated here—PPP3/calcineurin-dependent regulation of TFEB—is not unique to cardiac cells but represents a fundamental cellular mechanism conserved across multiple cell types. Indeed, PPP3/calcineurin-TFEB signaling has been characterized in diverse cellular contexts including epithelial cells (Medina et al., 2015), fibroblasts (Xia et al., 2024), and immune cells (Scientific aCo, Industrial Research IoMT, Chandigarh, India, 2018). Therefore, while the translational implications for myocarditis should be interpreted with appropriate caution, the mechanistic insights gained from this study provide a valuable framework for future investigations in cardiac-specific systems.

In conclusion, our results elucidated the mechanism of action of TAC-targeted regulation of TFEB and revealed the role of TAC in CVB3-targeted regulation of TFEB. This could provide a new intervention target for CVB3 infection-related diseases, which has important theoretical significance and application value.

## Data Availability

The original contributions presented in the study are included in the article/**Supplementary Material**. Further inquiries can be directed to the corresponding authors.
