# Supplementary material for: Tacrolimus inhibits CVB3-targeted regulation of TFEB by PPP3/calcineurin
Source: Front Cell Infect Microbiol. 2026 Jul 8;16:1826524. doi: 10.3389/fcimb.2026.1826524 (PMC13389937; doi:10.3389/fcimb.2026.1826524)
Supplement: Supplementary file 1 [file SupplementaryFile1.docx]

Figs.S1 Determination of TAC concentration. A-C. Western blot detection of different concentrations of TAC on TFEB expression.


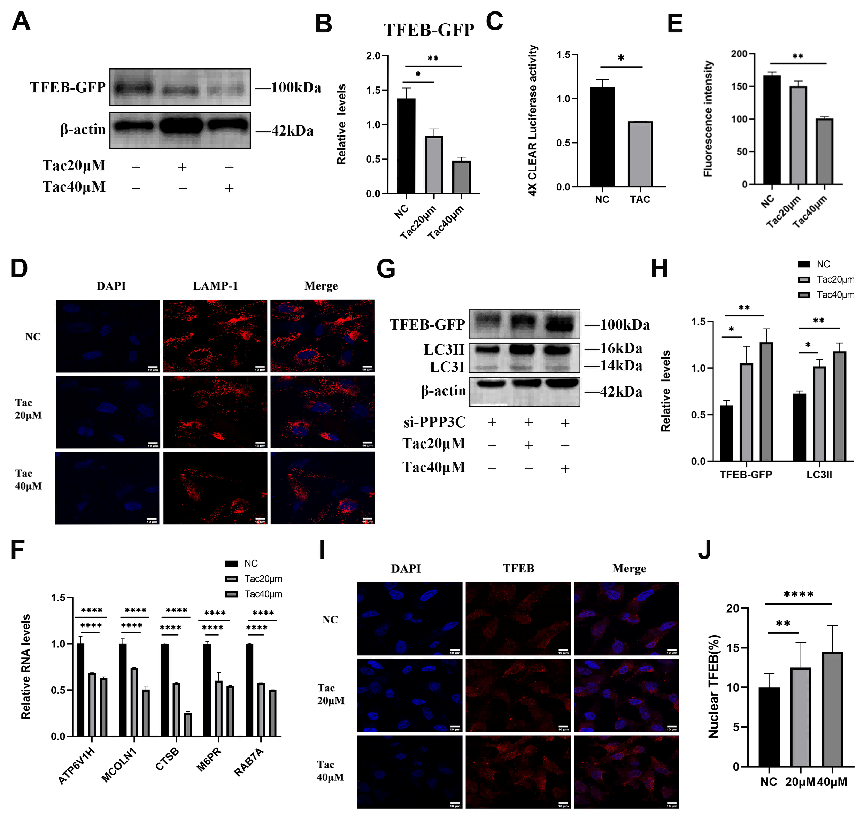
 Figs.S2 TAC inhibits TFEB-GFP by targeting PPP3/calcineurin. A-B. Western blot was performed to detect the expression level of TFEB-GFP. C. 4× CLEAR Luciferase reporter gene assay for TFEB-GFP transcriptional activity. D-E. Immunofluorescence detection of TFEB-GFP and LAMP-1 colocalization (or staining). Scale bar=25μm. F. The level of transcription of TFEB downstream target genes by RT-qPCR. G-H. PPP3C knocked down, western blot was performed to detect the expression levels of ∆60-TFEB and LC3II. I-J. PPP3C knocked down, then different concentrations of TFEB-GFP in the nuclear. Scale bar=10μm. n = 3-4 per group. Data are shown as the mean±SEM. * P<0.05, ** P<0.01, *** P<0.001.


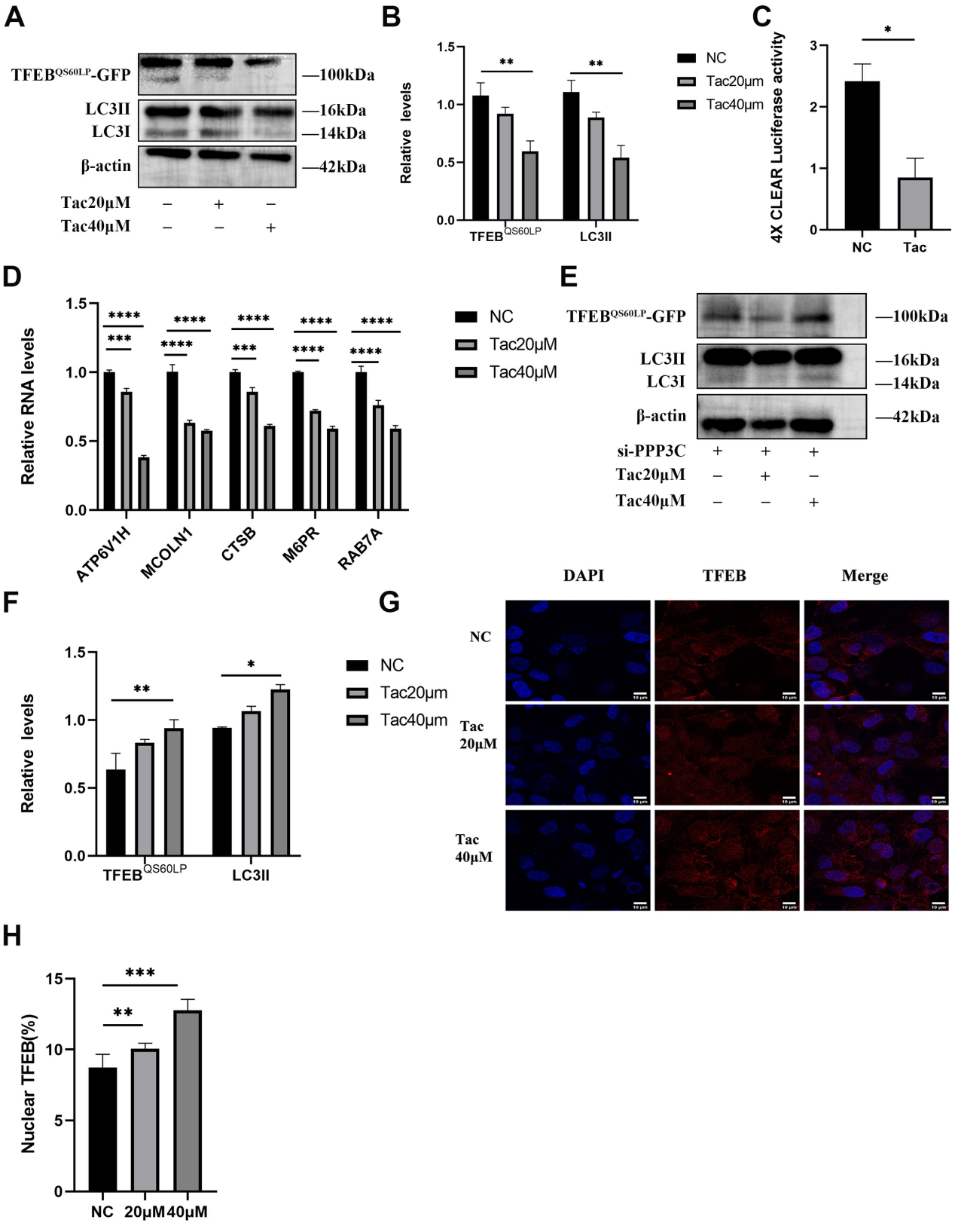
 Figs.S3 TAC inhibits ∆60-TFEB and TFEB^QS60LP^ by targeting PPP3/calcineurin. A-B. Western blot was performed to detect the expression level of TFEB^QS60LP^ and LC3II. C. 4× CLEAR Luciferase reporter gene assay for TFEB^QS60LP^ transcriptional activity. D. The level of transcription of TFEB downstream target genes by RT-qPCR. E-F. PPP3C knocked down, western blot was performed to detect the expression levels of TFEB^QS60LP^ and LC3II. G-H. PPP3C knocked down, then different concentrations of TFEB^QS60LP^ in the nuclear. Scale bar=10μm. n = 3-4 per group. Data are shown as the mean±SEM. * P<0.05, ** P<0.01, *** P<0.001.



 Figs.S4 A-B. Western blot was performed to detect the expression level of TFEB^QS60LP^ with CVB3 infection for 5h. C-D Western blot was performed to detect the expression level of TFEB^QS60LP^ with CVB3 and TAC infection. E-F. Immunofluorescence detection of TFEB^QS60LP^ staining with CVB3 and TAC infection. n = 3-4 per group. Data are shown as the mean±SEM. * P<0.05, ** P<0.01, *** P<0.001.
