# Supplementary material for: Tacrolimus inhibits CVB3-targeted regulation of TFEB by PPP3/calcineurin
Source: Front Cell Infect Microbiol. 2026 Jul 8;16:1826524. doi: 10.3389/fcimb.2026.1826524 (PMC13389937; doi:10.3389/fcimb.2026.1826524)
Supplement: Supplementary file 2 [file Table1.docx]

Table S1.Primer sequences used for quantitative PCR.

| Gene | Forward primer | Reverse primer |
| --- | --- | --- |
| ATP6V1H | CCCTGAAGAGAAGCAAGAGATG | TGCAGCATATCATCCACCATAG |
| MCOLN1 | GGAAAGCAGCTCCAGTTACA | GATGAGGCTCTGGAGGTTAATG |
| CTSB | GGACAAGCACTACGGATACAA | GTAGAGCAGGAAGTCCGAATAC |
| M6PR | CTCAGTGTGGGTTCCATCTTAC | GGGAAACTGCTCCATTCCTT |
| RAB7A  PPP3CA | CCTGGAGTCTTGGCCATAAAG  GCTGCCCTGATGAACCAACA | GAGAAGGTCCAAGTTCTGGTTC  GCAGGTGGTTCTTTGAATCGG |
| CVB3 VP1  CVB3 3D | ACTATGCAGACACGCCACGTTAAG  TCTAGGCTGATTGAGGCGTCCAG | GCATACCGCTTGGCACCTGAG  CCAACAGCACTACCAGTCACAACC |

Table S2. Sense and antisense siRNA sequences.

| Gene | | Sequence (5'to 3') |
| --- | --- | --- |
| PPP3C siRNA sense strand | | UCACAGAGAUGCUGGUAAATT |
| PPP3C siRNA antisense strand | UUACCAGCAUCUCUGUGATT | |
| Control siRNA sense strand | GUAUGACAACAGCCUCAAGTT | |
| Control siRNA antisense strand | CUUGAGGCUGUUGUCAUACTT | |
| TFEB siRNA sense strand | GCAUCAAGGAGUUGGGAAUTT | |
| TFEB siRNA antisense strand | AUUCCCAACUCCUUGAUGCTT | |
| Control siRNA sense strand | GUAUGACAACAGCCUCAAGTT | |
| Control siRNA antisense strand | CUUGAGGCUGUUGUCAUACTT | |
